# Encoding of Slowly Fluctuating Concentration Changes by Cockroach Olfactory Receptor Neurons Is Invariant to Air Flow Velocity

**DOI:** 10.3389/fphys.2019.00943

**Published:** 2019-08-07

**Authors:** Maria Hellwig, Alexander Martzok, Harald Tichy

**Affiliations:** Department of Neurobiology, Faculty of Life Sciences, University of Vienna, Vienna, Austria

**Keywords:** olfactory receptor neurons, ON and OFF responses, rate of concentration change, gain control, air flow velocity

## Abstract

The ON and OFF olfactory receptor neurons (ORNs) on the cockroach antenna display a high sensitivity for the rate at which odorant concentration changes. That rate of change acts as a gain control signal that improves the sensitivity of both ORNs for fluctuating concentration changes. By means of extracellular recording techniques, we find in both types of ORNs an increased gain for the rate of concentration change when the duration of the oscillation period increases. During long-period oscillations with slow concentration changes, the high gain for the rate of concentration change improves the ORNs ability to detect low rates of concentration changes when the fluctuations are weak. To be useful in plume tracking, gain control must be invariant to the air flow velocity. We describe that raising the level of the flow rate has no effect on the ON-ORN responses to concentration changes down to rates of 2%/s, but exerts a slight increase on the OFF-ORN response during these extremely low rates. At 4%/s, however, the OFF-ORN response is also unaffected by the flow rate level. The asymmetry corresponds with a generally higher sensitivity of the OFF-ORN to concentration changes. Nevertheless, the gain of both ORNs for the concentration rate change is robust against the air flow velocity. This makes possible an instantaneous analysis of the rate of concentration change for both directions of change by one or the other ORN. Therefore, the ON and OFF ORNs are optimized to encode concentration increments and decrements in a turbulent odorant plume.

## Introduction

The primary objective of this study was to determine what effect the rate of the air flow carrying the odorant across a cockroach’s antenna has upon the activity of olfactory receptor neurons (ORNs). The work leading up to this study began with the identification of pairs of ORNs in a structurally identifiable sensillum type which respond antagonistically to the same change in odorant concentration. In this way, concentration increments and decrements are encoded by excitatory signals. During slow and continuous concentration changes, both types of ORNs not only signal the moment-to-moment succession of odorant concentrations but also the rate at which concentration changes (Burgstaller and Tichy, 2011, 2012; Tichy and Hellwig, 2018). Furthermore, the rate of concentration change modulates the gain of responses for fluctuations in the odorant concentration. When odor concentration changes slowly, both ORN types improve the gain for the rate of change at the expense of the gain for the instantaneous concentration. This suggests that the ORNs are optimized to detect minute changes in odorant concentration, even if they persist in one direction.

The instantaneous odorant concentration and its rate of change are two independent variables because each can be changed without producing a change in the other. Concentration has been very often manipulated in insect olfactory research, but the rate of concentration change has received less attention. In most studies, odorants were applied as transient concentration pulses. The rate of concentration change at the onset of the odorant pulse was not measured and its possible effect on the response magnitude was not determined. Stimulus-response functions were based on the mean pulse concentration and the mean discharge of the pulse period. According to this concept, the effect of changes in the velocity of odorant pulses was studied in two types of ORNs on the antennae of *Drosophila* (Zhou and Wilson, 2012). Pulse velocity was changed by varying the amount of volume of the odorant-loaded air that was delivered during the pulse period. If pulse concentration was kept constant, the ORNs responses were invariant to changes in the pulse flow rate. Unfortunately, the phasic component of the response as a possible candidate for encoding the rate of concentration change was neglected. The ORNs were regarded as “transducers of concentration,” responding to given concentration pulses independently of the pulse velocity (Zhou and Wilson, 2012).

In contrast, the ON and OFF ORNs of the cockroach may be transducers of the “rate of concentration change,” with an additional dependence on the instantaneous concentration at which the change occurs. The observation of potentially different transduction mechanisms is based, however, on different methods of delivering the odorant. While concentration pulses rapidly immerse the whole sensillum into the stimulus concentration, slow concentration changes gradually increase the concentration at the sensillum surface. For example, it requires 10 s to increase the concentration from 0 to 50% at a rate of 5%/s, but 25 s at a rate of 2%/s. Therefore, when increasing the concentration on the sensillum surface at a rate of 5%/s, an instantaneous concentration of 25% is reached 5 s after the onset of the concentration increase, and after 12.5 s at a rate of 2%/s. Furthermore, the higher the flow rate, the greater is the volume of odorant-loaded air that passes over the antenna. Thus, the same gradual concentration increase delivered at a higher flow rate involves a greater quantity of molecules arriving per unit time on the sensillum surface.

In natural foraging environments, wind speed and the direction of wind flow are the most important factors affecting odorant concentration. We can expect that animals tracking a turbulent odorant plume discriminate flow velocity invariant concentration changes. Flying insects use wind information and visual feedback to efficiently track an odor plume, but walking lobsters and crabs perform true chemotaxis, whereby the temporal analysis of odorant pulse features guides orientation along plumes. In behavioral studies, lobsters use a spatial gradient in pulse size and shape to locate the odorant source (Moore and Atema, 1991). The spatial distribution of the pulse onset slopes and the correlated pulse amplitudes provide the strongest gradient pointing to the source. In such an “odor landscape,” the peak height of pulses and the onset slope of these peaks increase with decreasing distance to the odor source (Moore and Atema, 1991; Zettler and Atema, 1999). Electrophysiological recordings provide evidence for the existence of “pulse slope detectors” on chemoreceptors of the lateral antennules of the American lobster (Zettler and Atema, 1999). In the cockroach, the ON and OFF ORNs on the antennae meet the requirements of detectors for the upward and downward rate of change of the food odor concentration (Tichy and Hellwig, 2018). If the rate of concentration change is truly a fundamental aspect of insect orientation in an odorant plume, information about the concentration rate should be robust across a range of different flow velocities.

Any study attempting to evaluate the individual effects of the instantaneous concentration, its rate of change and the flow velocity of the odorant-carrying air steam must satisfy two special requirements. First, it must utilize techniques and procedures to regulate, control and monitor all three stimulation variables simultaneously. Second, it must ensure that data analysis recognizes potential confusion when unscrambling the effect of interrelated variables. The present study incorporates both these requirements. The first is met by a dilution flow olfactometer that enables producing olfactory stimuli of precise air pressure, flow velocity, as well as concentration and its rate of change. The second is met by utilizing software that relates the responses of the ORNs to different combinations of the three variables and then estimates how much each variable contributes to the response.

The above considerations yield two readily testable predictions. Both predictions are critical in evaluating the function of the ON and OFF ORNs as “concentration rate detectors.” Each involves two exclusive statements.

The first prediction is that variation in the level of the volume flow rate has no effect on ORN responses to slow and continuous concentration changes. Thus, the ORNs would be able to detect changes in the odorant concentration (the ratio between molecule number and air volume) regardless of the volume size, the absolute number of molecules involved in the concentration change, the rate of arrival at the antenna or the rate of air flow. Alternatively, the response to equal rates of concentration change would increase with increasing flow rate level due to the increasing absolute number of odorant molecules arriving at the sensillum.

The second prediction is that the level of the volume flow rate does not affect the increased gain for the rate of concentration change with increasing duration of the oscillation period. Alternatively, gain control varies with the flow-rate level. Then the concentration rate no longer needs to be considered as a gain control signal.

## Materials and Methods

### Preparation and Recording

Adult male cockroach (*Periplaneta americana*) were anesthetized with CO_2_, placed on their dorsal surface in a closely fitted holder and fixed with strips of Parafilm wrapped around the holder. One antenna was kept in a forward position by cementing it onto a ledge that extended from the holder. Action potentials were recorded extracellularly between two electrolytically sharpened tungsten wires. The reference electrode was placed lengthwise in the tip of the antenna and the recording electrode was inserted into the base of the sensillum. Impulses were amplified and band filtered (0.1–3 kHz), passed through a 1401plus A-D converter (Cambridge Electronic Design) and fed into a PC. The digitized impulses, the voltage output of the electronic flow meters and the PID signal were displayed on-line on a monitor, stored on a hard disk and analyzed off-line using Spike2 software.

### Odorant Stimulation

The odor of lemon oil is known to activate antennal ORNs and antennal lobe neurons (Sass, 1978; Selzer, 1981, 1984; Boeckh et al., 1990; Zeiner and Tichy, 2000). It contains a number of odor compounds of different chemical classes (Günther, 1968; Shaw, 1979). Based on its reproducibility, synthetic lemon oil (Roth, D ∼ 0.85, Art. 5213.1) rather than natural fruits were used as a standardized fruit odorant stimulus.

Stimulation was provided using a dilution flow olfactometer (Prah et al., 1995; Burgstaller and Tichy, 2011, 2012). Pre-cleaned and pre-dried compressed air from a laboratory line was passed through an adsorption drier (DPS 1-8A; Filtrations-Separations-Technik, Essen, Germany). The stream was then divided into two equal-sized streams *A* and *B*. Their flow rates were regulated by manual needle valves in series with calibrated flow meters of the Rotameter type. Stream *A* was bubbled through small holes in polyethylene tubing anchored at the bottom of a 25-l tank containing 100 ml of the undiluted liquid odor of lemon oil. Stream *B* was led through an empty tank of the same design and remained clean. After emerging from the tanks, the air streams passed through electrical proportional valves (Kolvenbach KG, KWS 3/4) and electronic flow meters (AWM 3000, Honeywell). The sinusoidal concentration changes were produced by shifting the phase of the valves’ control voltages (D-A converter, 1401plus, Cambridge Electronic Design) by 180°. Thus, the total volume flow rate of both air streams was held constant as the underlying odor/clean-air ratio was varied in a sinusoidal manner. This ratio was regulated by the output sequencer function of the data acquisition software [Spike2, v.3.18; Cambridge Electronic Design (CED), Cambridge, United Kingdom], using a self-written sequencer script. A feedback linearization, which integrated the voltages used to control the proportional valve with those received from the flow meters, counteracted any deviations of the flow rate set by the output sequencer. For stimulation, the mixed air stream emerged from a 7-mm-diameter nozzle at a distance of 10 mm from the recording site. The air around the antenna was continually removed by a suction tube at a speed of 2 m/s.

**FIGURE 1:**
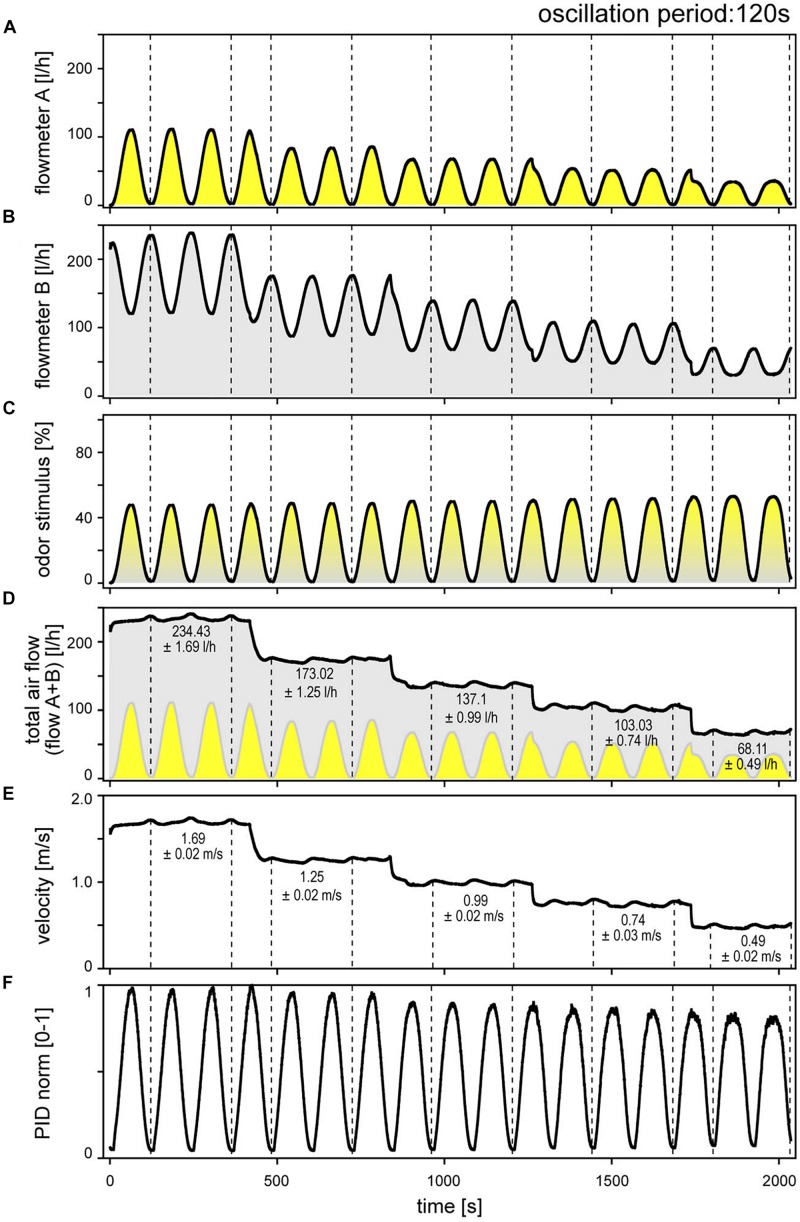
Sinusoidal concentration changes at constant period of 120 s, delivered at five different, stepwise reduced air flow velocities. **(A)** Flowmeter *A* signals the time course of the odor-saturated air stream controlled by proportional valve *A*. **(B)** Flowmeter *B* signals the time course of the clean air stream controlled by proportional valve *B*. The control voltages of both valves were 180° phase-shifted. In order to maintain constant amplitude of the sinusoidal concentration changes when the flow velocity was decreased, the flow rates of both air streams were reduced accordingly. **(C)** Time course of sinusoidal concentration changes determined by the mixing ratio of odor-saturated air in clean air and expressed as percentage of saturated air. **(D)** Time course of summed flow rates of the odor-saturated air stream *A* (yellow oscillations) and the clean air stream *B* (gray oscillations, inverse to yellow oscillations); averaged values were determined for the periods indicated by dotted lines. **(E)** Time course of the flow velocity of the mixed air stream, calculated from the total flow rate of the two air streams in panel **(D)**. Periodic low-amplitude fluctuations in air flow velocity reflect fluctuations in the total air flow rate; they had no effect on ORN responses. Averaged values were determined for the periods indicated by dotted lines. **(F)** Normalized time course of the sinusoidal PID signal corresponds with the flowmeter signal in panel **(C)**; note slow decrease in amplitude with decreasing flow velocity.

The digitized output voltage of the electronic flow meters, calibrated by the manufacturer for flow rate, was used to monitor the flow profiles of the two individual air streams and of the mixed air stream representing the odor delivery during stimulation. Figure 1 illustrates an example for a 120-s oscillation period performed at five different volume flow velocities between 0.49 and 1.64 m/s. The flow-rate ratios of the oscillating odor-saturated air stream (Figure 1A) to the oscillating clean air stream (Figure 1B) determined the oscillating concentration changes, indicated as percentage of the saturated air stream in the mixed air stream (Figure 1C). The velocity of the odor stimulus was varied by regulating the flow rates of the input air streams *A* and *B* with the manual needle valves. Modifying the flow rates changed the oscillation amplitudes. In order to set the velocity of the mixed air stream to the required values without changing the amplitude of the concentration oscillations, the flow rates of the two air streams were adjusted via the proportional valves. Thus, the oscillations of the odor-saturated air stream (*A*) were confined to the lower half of the flow rates and, with each step-wise decrease in velocity, the amplitude of the flow rate decreased (Figure 1A). The oscillations of the clean air stream (*B*) initially occurred in the high range of flow rates, and then stepped downward to the lower flow rates, thereby spanning smaller amplitude flow rates (Figure 1B). The resulting amplitude of the concentration oscillation was 50% (Figure 1C). Plots of the summed flow-rate profiles of both air streams served to verify that the total output flow rate of the mixed air stream is constant at each velocity (Figure 1D). Nonetheless, the total flow rate indicated low-amplitude, slow fluctuations which appeared to be in phase with the flow-rate oscillations of the two air streams. Average values for these changes in the total flow rate attained a maximum of 0.7 l/h. The direction of change in the ORNs’ activity and the fluctuations in the total air flow rate were not correlated. No faster fluctuations or random distribution of fluctuations in the flow rate were apparent. The fluctuations might be due to the inherent flow characteristics of the valves, taking into account the effects of piping. The total air flow rate (Figure 1D) that passes the cross-section area (diameter 7 mm) of the output nozzle per second specifies the velocity of the stimulating air stream (Figure 1E). Mean velocity values were calculated for periods indicated by dotted lines, which span two oscillation periods (Figure 1E); the standard deviations are low (±0.02 m/s).

A photoionization detector (200A miniPID, Aurora Scientific) was used to verify that mixing of the slowly oscillating, changing flow rates of the two air streams actually produces slowly oscillating concentration changes at the different air velocities (Figure 1F). Flow meters controlled the timing and amplitude of the concentration oscillations within the delivery tubes. At the same time, the PID needle was positioned between the output nozzle and the antenna. The PID signal is specified as being proportional to the concentration of the compound entering the flow-through detection cell. This signal is reproducible for a given compound and pressure. As the PID output voltage was not calibrated with a reference gas, the absolute odorant concentration was not measured. The PID output voltages were normalized such that the maximum value obtained in an experiment was arbitrarily set to the value “1.” The time course of the oscillating PID signal with maxima and minima values (Figure 1F) matches with those of the concentration oscillations obtained by the electronic flow meters (Figure 1C). However, the amplitude of the PID signal slightly decreased with decreasing air flow velocity. The same decrease is not shown by the flow meter signal. A specific feature of the concentration measurement was the positive pressure from the nozzle and the negative pressure at the sample pump. This pressure difference depends on the volume flow velocity. No systematic attempt was made to determine whether the divergence between the oscillating PID signal and the oscillating the flow-meter signal affects the oscillating activity of the ON and OFF ORNs. However, the gain for the rate of concentration during oscillating changes appears to decrease slightly with decreasing air flow velocity, as revealed by plotting impulse frequency of the ON and OFF ORNs as function of both the rate of change and velocity. As the decrease in air velocity is correlated with the decrease in the PID signal, one may argue that the effects assigned to the decrease in the flow velocity would indeed be due to the decrease in the amplitude of the concentration oscillations. Since a possible effect becomes apparent only at a low velocity of 0.45 m/s (Figure 1D) and a slow rate of change of 3.5%/s (Figure 1E), no systematic attempt was made to determine the extent to which the divergence between the PID signal and the flow-meter signal affects gain control of the ORNs. If it does, the effects were not obvious.

### Recordings

Electrodes were electrolytically sharpened tungsten wires. The reference electrode was placed in the tip of the antenna; the recording electrode was inserted into the base of the sensillum. All recordings were taken from *swC* sensilla, which are single-walled basiconic sensilla (Schaller, 1978; Altner et al., 1983; Hinterwirth et al., 2004; Tichy et al., 2005). Impulses were amplified and filtered (0.3–1 kHz), passed through a 1401plus A-D converter (Cambridge Electronic Design, United Kingdom) and fed into a PC. The digitized action potentials and the voltage output of the electronic flow meters were displayed on-line on a monitor, stored on a hard disk and analyzed off-line using the Spike2 software. Spike waveform parameters were extracted and sampled to form templates. Detected spikes were offered to the template matching system in order to create or modify the templates. Each spike was compared against the templates, and each time a template was confirmed it was added to the template by overdrawing (Figures 3G,H). Adding a spike to a template may change template configuration. The template boundaries displayed homogeneity of classification without a gradual change from one class to the other.

## Results

### Identification

**FIGURE 2:**
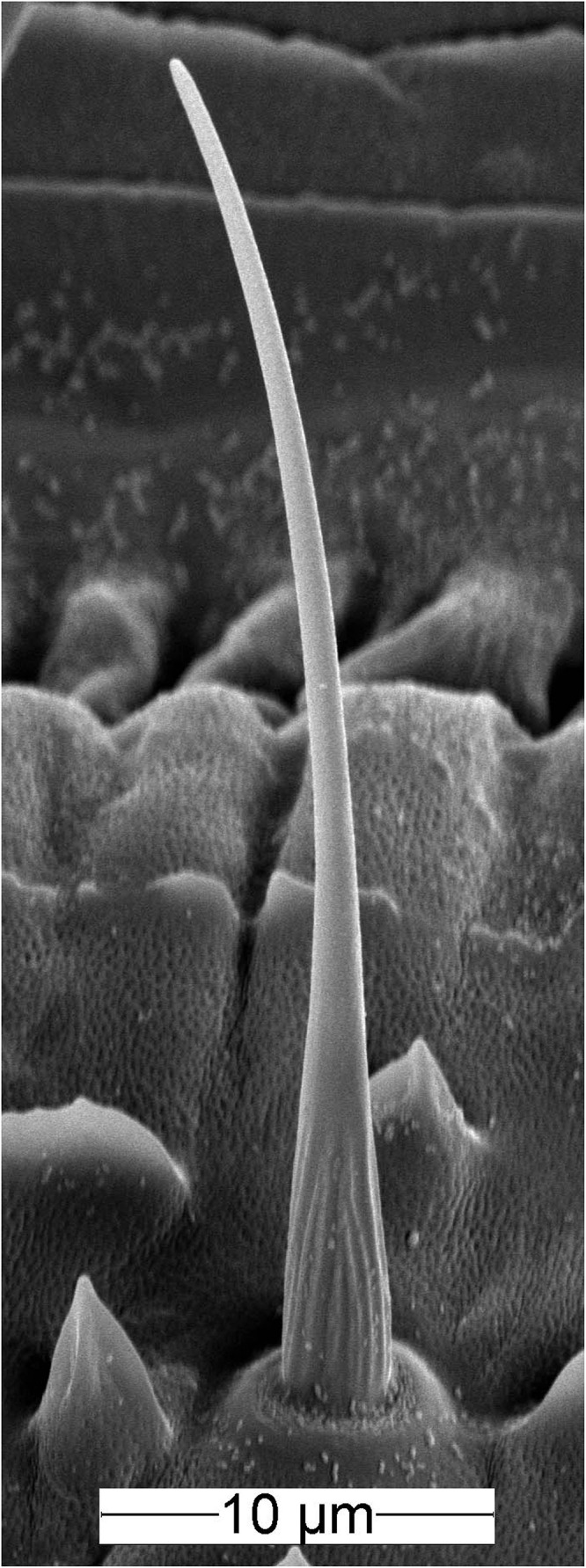
Scanning electron micrograph of the olfactory sensillum on the cockroach antenna containing the ON and OFF ORNs. The sensillum has a slender, hair-like form with a slightly curved tip. The basal part of the shaft wall is grooved, the distal part is smooth and perforated by pores.

**FIGURE 3:**
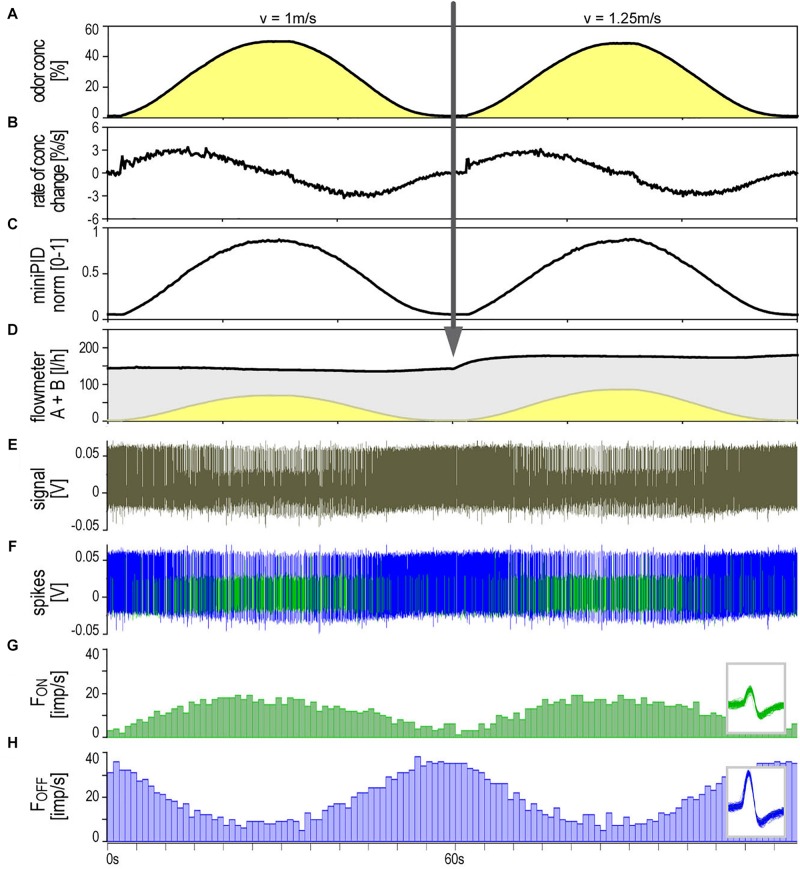
Simultaneously recorded single sensillum responses of a pair of ON and OFF ORNs. Stimulation consisted of slow and continuous upward and downward changes of the concentration of lemon oil odorant delivered at two different air flow velocities. Arrow indicates change in air flow velocity from 1 to 1.25 m/s. **(A)** Time course of instantaneous odorant concentration. Duration of oscillation periods: 60 s. **(B)** Time course of the rate of concentration change. The maxima and minima of the oscillating rate of concentration change are in advance of the maxima and minima of the oscillating instantaneous odorant concentration (vertical dotted line). The noisy signal results from the slow rate of concentration change in a narrow range of ±3.5%/s. **(C)** Time course of odorant concentration measured with a photoionization detector (miniPID). The signal values were normalized to the maximum value obtained in each experiment and set to 1. The concentration maxima and minima indicated by the oscillating flowmeter *A* signal **(A)** correspond with the concentration maxima and minima of the PID signal. **(D)** Time course of the summed flow rates of the odor-saturated air stream measured with flowmeter *A* (yellow oscillations) and the clean air stream measured with flowmeter *B* (gray oscillations, inverse to yellow oscillations); the summed flow rates of both air streams were used to calculate the air flow velocity of the olfactory stimulus. **(E)** Extracellularly recorded activity of the ORNs. Both types discharge continuously during the concentration cycles. The OFF ORN typically generates greater impulse amplitudes than the ON ORN. **(F)** Off-line sorted action potentials of the ORNs obtained by spike detecting and template matching techniques using Spike2 software (Cambridge Electronic Design, United Kingdom). Green impulses originate from the ON ORN, blue impulses from the OFF ORN. **(G,H)** Instantaneous impulse frequency of the ON and OFF ORNs, respectively; bin width, 0.2 s. *Insets* action potentials classified by matching the shape of each action potential against shape templates. Template windows show template boundaries of spike waveforms from the two ORNs. *F* impulse frequency, *v* velocity.

The ON and OFF ORNs share a specific single-walled sensillum type which arises as a hair-like structure from a ring-shaped socket (Figure 2). Characteristic features are longitudinal grooves in the surface of the basal part of the shaft and a slender tapering tip. Neuroanatomical and electrophysiological studies show that these sensilla are located on the distal and proximal margins of each of the 120–180 antennal segments. They make up about 6% of the olfactory sensilla in the male cockroach (Schaller, 1978; Burgstaller and Tichy, 2012; Tichy and Hellwig, 2018). Inserting the tip of a needle electrode into the sensillum base enables recording the action potentials of both ORN types at the same time. The OFF ORN typically displayed larger impulse amplitudes than the ON ORN. The clear differences in size and form of the impulses facilitated final identification of the different OFF ORNs by their antagonistic responses to slowly oscillating changes in odor concentration. A typical example is illustrated in Figure 3. Figure 3A shows the time course of the odorant concentration oscillating at a period of 60 s, and Figure 3B the corresponding rate at which concentration oscillates. Figure 3C indicates the time course of the miniPIDsignal and Figure 3D the flow rates of both the odor-saturated and the clean air stream. Increasing the concentration of the odorant of lemon oil raises the impulse frequency in the ON ORN (Figure 3E) and lowers it in the OFF ORN (Figure 3F). Correspondingly contrary effects are elicited by decreasing the odorant concentration. As indicated by the time-histograms in Figures 3G,H, the ON-ORN’s impulse frequency peaks just before the maximum instantaneous concentration, and that of the OFF-ORN just before the concentration minimum. The rate of concentration change, which is ahead of the oscillating instantaneous concentration, clearly also determines the activity of both ORNs.

### Gain of Response for the Rate of Concentration Change

Of the 40 pairs of ON and OFF ORNs on which oscillating concentration changes were tested, only six pairs qualified for this study: those whose firing rates continued undiminished for the duration of the experiment. In most recordings, the amplitudes of the action potentials tended to decrease with time. The cause of this diminution is unclear. The shape of the electrode, its depth and position relative to the two ORNs surely differed to some extent with every insertion.

**FIGURE 4:**
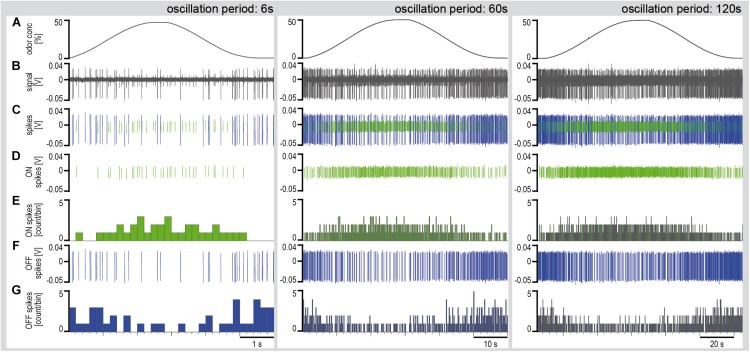
Simultaneously recorded single sensillum responses of a pair of ON and OFF ORNs to oscillating concentration changes of the lemon oil odorant with periods of 6, 60, and 120 s. Air flow velocity: 1.69 m/s. **(A)** Time course of instantaneous odorant concentration. **(B)** Electrical activity of the ON and OFF ORNs. Both types discharge continuously during the concentration cycles. The large impulse amplitudes are from the OFF ORN. **(C)** Off-line sorted action potentials of the ORNs obtained by spike detecting and template matching techniques using Spike2 software (Cambridge Electronic Design, United Kingdom). Green impulses are from the ON ORN, blue impulses from the OFF ORN. **(D)** Discriminated impulses of the ON ORN. **(E)** Instantaneous impulse frequency of the ON ORNs; bin width, 0.2 s. **(F)** Discriminated impulses of the OFF ORN. **(G)** Instantaneous impulse frequency of the OFF ORNs; bin width, 0.2 s. *F* impulse frequency, *V* voltage.

**FIGURE 5:**
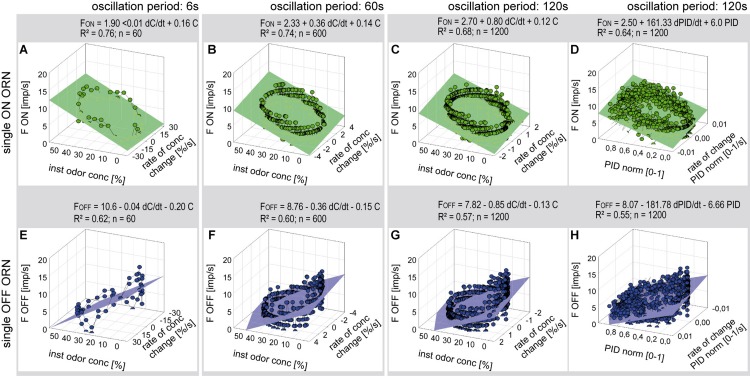
Responses of a single ON ORN **(A–D)** and a single OFF ORN **(E–H)** during oscillating concentration changes plotted as a function of the instantaneous concentration and the rate of concentration change. Instantaneous concentration and its rate of change were measured in panels **(A–C, E–G)** with the flowmeter *A*, in panels **(D,H)** with the miniPID. Duration of oscillation period: 6 s in panels **(A,E);** 60 s in panels **(B,F);** 120 s in panels **(C,D,G,H).** Multiple regressions utilizing 3-dimensional planes *F* = *y*_0_ + *a* d*C/*d*tC* + *b C*; where *F* is the impulse frequency, *y*_0_ the height of the regression plane) were calculated to determine the gain of response for the rate of concentration change (*a* slope) and the instantaneous concentration (*b* slope). Note that the sign of the concentration axis in panels **(A–D)** is oriented in different direction than in panels **(E–H).** *R*^2^ coefficient of determination, *n* number of points used to calculate regression plane.

Series of constant-amplitude oscillating concentration changes were tested with periods of 6, 60, and 120 s (Figure 4). The rate of change averaged 30%/s during the 6-s period, 3.5%/s during the 60-s period and 2%/s during the 120-s period. To estimate the dependence of the ORNs on the instantaneous concentration and the rate of concentration change, impulse frequency was plotted as a function of both parameters (Figure 5). The impulse frequency curves approached closed figures resembling Lissajous figures, which are formed when two parameters oscillate with the same frequency and are plotted one as a function of the other. These shapes depend on the ratio of the frequencies of the two oscillations, the ratio of their amplitudes and their phase differences.

Multiple regressions (*F* = *y*_0_ + *a* d*C*/d*t* + *b C*; where *F* is the impulse frequency and *y*_0_ the intercept of the regression plane, with the *F* axis reflecting the height of the regression plane) were calculated to determine the gain of responses for the rate of concentration change (*a* slope) and the instantaneous concentration (*b* slope). The sign of the regression slopes is positive for the ON ORN (Figures 5A–D) and negative for the OFF ORN (Figures 5E–H), i.e., an increase in both the instantaneous concentration and its rate of change raises the impulse frequency in ON ORN and lowers it in the OFF ORN. Accordingly, during oscillating concentration change, the ON ORN’s impulse frequency is high when the instantaneous concentration is high and even higher the faster the concentration rises through the higher values (Figures 5A–D). Conversely, the OFF ORN’s impulse frequency is low when the instantaneous concentration is low and even lower the faster the concentration falls through the lower values (Figures 5E–H). Furthermore, slope steepness varies with the duration of the oscillation period. In both ORNs, sign ignored, the gain for the rate of change tends to be lower during short oscillation periods and higher during long periods. In the examples shown in Figure 5, the ON-ORN’s gain for the rate of concentration change was 0.01 imp/s per %/s at a period of 6 s, 0.36 imp/s per %/s at 60 s, and 0.80 imp/s per %/s at 120 s. In the OFF ORN, the gain for the rate of concentration change was –0.04 imp/s per %/s at 6 s, –0.36 imp/s per %/s at 60 s and –0.85 imp/s per %/s at 120 s.

To confirm that the periods and amplitudes of the concentration oscillation correspond to the settings, the time course of the odorant stimulus was measured with a miniature photoionization detector (miniPID). As illustrated in Figure 3, the time course of the normalized PID signal was synchronous to the flowmeter signal, confirming that the dilution flow olfactometer precisely controlled the odorant stimulus. The relationship between the ORNs’ responses, the instantaneous PID signal and its rate of change revealed the same double dependence as obtained with the flowmeter signal (Figures 5D,H). In view of this good correspondence, graphs of PID signals used for estimating stimulus-response relationships are not shown.

### Gain of Response at Different Air Flow Velocities (Volume Flow Rates)

Figure 3 illustrates the activity of an ORN pair during two subsequent 60-s oscillation periods tested at different constant air flow velocities (1 and 1.25 m/s). At both velocities, the oscillating frequency curves were smooth, their phase advance on the oscillating concentration curve was present, and the signal-to-noise ratio was high. The question was whether the air velocity affects the response magnitude of both ORNs and gain control. Air flow velocity was controlled by the volume of air per unit time flowing out of the nozzle of the odorant delivery system at a distance of 10 mm from the recording site. Since the effects of flow velocity on ORN responses are comparable only for equal concentration changes, the obvious procedure was to record the responses to constant period oscillations for every volume flow rate level. On each ORN, five different flow rate levels were tested between 0.49 to 1.69 m/s.

**FIGURE 6:**
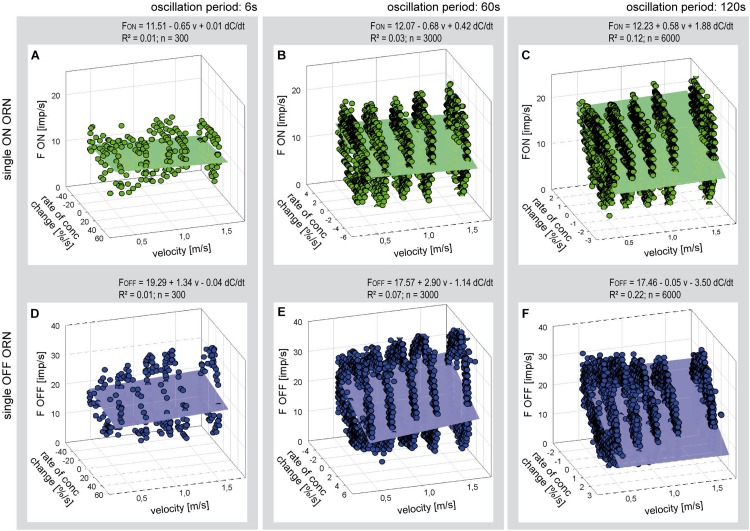
Responses of a single ON ORN **(A–C)** and a single OFF ORN **(D–F)** during three different periods of oscillating concentration changes plotted as a function of the rate of concentration change and air flow velocity. Duration of oscillation period: 6 s in panels **(A,D)**; 60 s in panels **(B,E)**; and 120 s in panels **(C,F).** Multiple regressions utilizing 3-dimensional planes (*F* = *y*_0_ + *a v* + *b* d*C/*d*t*; where *F* is the impulse frequency, *y*_0_ the height of the regression plane) were calculated to determine the effect of velocity (*a* slope) on the responses to the rate of concentration (*b* slope) during oscillating changes. Note that the sign of the rate of concentration axis in panels **(A–C)** is oriented in different direction than in panels **(D,E)**. *R*^2^ coefficient of determination; *n* number of points used to calculate regression plane, *v* velocity.

For each oscillation period, impulse frequency of the six ON and OFF ORNs was plotted as a function of both the level of flow rate and the rate of concentration change. Figure 6 is an example of a simultaneously recorded pair of ORNs. The oscillating impulse frequencies form elliptic Lissajous curves as illustrated in Figure 5. Multiple linear regressions were used to estimate the dependence of the oscillating impulse frequencies on the flow rate level (*a* slope) and the rate of concentration (*b* slope) for each oscillation period. The horizontal orientation of the axis representing the effect of flow velocity as well as the very low values of the coefficient of determination (*R*^2^ < 0.2, *n* = 6.000) indicate that the regression planes failed to describe in both ORN types a dependence of the responses to the rate of concentration change on the air flow velocity.

**FIGURE 7:**
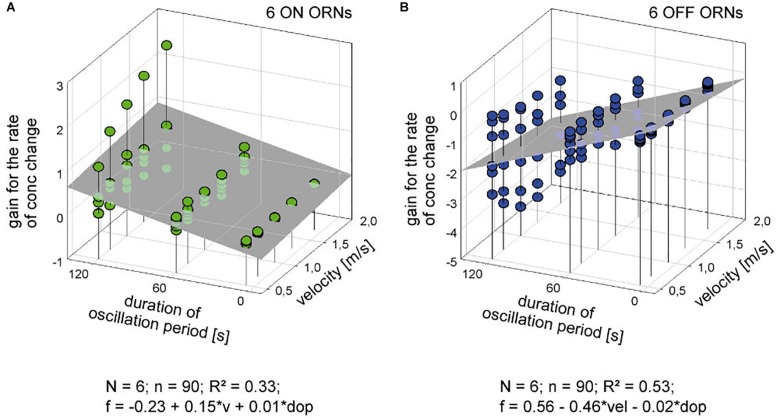
Gain for the rate of concentration change of six pairs of ON **(A)** and OFF ORNs **(B)** plotted as a function of the oscillation period and the volume flow velocity. Multiple regressions utilizing 3-dimensional planes (*f* = *y*_0_ + *a v* + *b dop*; where *f* is the gain of responses for the rate of concentration change, and *y*_0_ the height of the regression plane) were calculated to determine the effect of velocity (*a* slope) on the gain of responses for concentration oscillations with different periods (*b* slope). *R*^2^ coefficient of determination; *N* number of ORNs; *n* number of points used to calculate regression plane; *dop* duration of period; *v* velocity.

The poor coefficient of determination values may partly reflect the fact that the impulse frequency values of both ORNs cover a large part on the frequency scale. This is because the ORNs simultaneously depend on the instantaneous concentration and its rate of change. The effect of the instantaneous concentration will be reduced by plotting the gain values for the rate of concentration change rather than the oscillating impulse frequency as a function of both the air flow velocity and the oscillation period. This expectation is largely confirmed in Figure 7. The variations in the gain values appear to depend on the duration of the oscillation period: with increasing duration the deviations of the gain values from the regression plane increase. The regression slopes indicate a gain increase of the ON ORN by 0.15 (imp/s)/(d*C*/d*t*) for each 1 m/s increase in air velocity (Figure 7A); the corresponding values for the OFF ORN are an increase by –0.46 (imp/s)/(d*C*/d*t*) per 1 m/s (Figure 7B). (The negative values for gain reflect the downward direction of the concentration change yielding a rise in OFF-ORN impulse frequency). To increase the gain of the ON ORN by 1 (imp/s)/(d*C*/d*t*), the flow velocity must be increased by 6.6 m/s, and by 2.2 m/s for the OFF ORN. However, the moderate values of *R*^2^ (0.33 and 0.53 for the ON and OFF ORN, respectively) indicate that not more than 50% of the variance in the OFF-ORN’s gain can be explained by the flow velocity. The remaining 50% may be attributed to an inherent variability. Note that the variation of gain is highest for the 120-s oscillation period (Figure 7). At this long period, concentration changes at a mean rate of as low as 2%/s need 60 s to get from 0 to 50%, and 60 s to go back from 50 to 0%. Minute concentration fluctuations may produce low-frequency fluctuations in both ORNs. Nonetheless, as the gain for the rate of change increases with increasing duration of the oscillation period and increasing flow velocity, the sensitivity for the rate of change is not diminished but instead improved at slow changes and fast velocities.

## Discussion

Insect olfaction is usually assumed to reflect the perception of the odorant by ORNs, but it is largely dependent on the air flow across the antenna. This dependency is described in various olfactory orientation experiments (Atema, 1996; Vickers, 2000; Weissburg, 2000; Webster and Weissburg, 2001; Keller and Weissburg, 2004; Willis and Avondet, 2005; Willis et al., 2008; Page et al., 2011; Reidenbach and Koehl, 2011). The data on odorant plume structure has led to testable predictions on orientation strategies. These predictions focus on the intermittent mode of the chemical signal as well as the spatial and temporal concentration patterns that is critical for mediating upwind flight and casting behavior in several species of moths (Vickers, 2006). The onset slopes of odorant pulses, indicating a concentration increase at a particular rate, were only rarely considered as a factor in determining orientation behavior. Importantly, the pattern of the pulse onset slopes and pulse amplitudes form a spatial gradient. These point toward the plume source better than other dynamic parameters (Moore and Atema, 1991; Zettler and Atema, 1999). Lobsters use the temporal and spatial distribution of odorant signals to locate the sources (Moore and Atema, 1991). Cockroaches, however, have lifestyles and feeding ecologies quite different from those of lobsters. Nonetheless, as ground dwellers they will also use temporal and spatial pulse parameters during orientation along an odorant plume. Unfortunately, we lack detailed knowledge about the sensory mechanism underlying plume tracking to initially large odorant sources and their less disrupted plumes. Rapid and accurate orientation movements require unambiguously detecting the concentration and its rate of change at various air flow velocities. The greater the velocity, the greater is the flow rate which is defined as the amount of air volume flowing across an area per unit of time. An increase in the flow rate of a dispersing air volume at constant concentration does not lead to any change in the number of molecules per unit volume (viz. the ratio between molecule number and air volume), but does increase the absolute number of molecules delivered per unit time. ORNs acting as “pulse slope detectors” must assess the rate of concentration change of the odorant-loaded air, independently of the absolute number of molecules involved in the change, the odorant-loaded air volume or its flow rate.

In the context of the present study, the two predictions pointed out in the Introduction did hold to a remarkable degree. In regard to the first prediction, the response of the ON and OFF ORNs to oscillating concentration changes with a period of 120 s and a mean rate of 2%/s increases slightly by increasing the air flow velocity from 0.49 to 1.69 m/s. At a mean rate of 3.5%/s and a 60 s oscillation period, however, the flow velocity has no effect on ORN responses. Right down to these slow rates, the response of the ORNs is modulated by changes in the air stream concentration independently of the absolute number of molecules, the air volume involved in the concentration change, the rate of arrival of the odorant molecules at the antenna or the rate of air flow. The second prediction also holds in the above results. That is, the gain for the rate of concentration change is invariant to the flow-rate level. When the odorant concentration oscillates slowly with long periods, the cue would simply be that the ORN discharge rates begin to change at all. Because of the high gain at slow change rates, cockroaches will receive creeping changes in concentration, even if they persist in one direction. The mechanism underlying ORN gain control and the robustness of gain control against changes in odorant flow rates in the air stream is unclear.

The ability of the ON and OFF ORNs to correctly identify slow rates of concentration change despite variations in the volume flow rate agrees with the flow-rate invariant responses to concentration pulses in *Drosophila* ORNs (Zhou and Wilson, 2012). Nonetheless, there are differences in the stimulation technique, the method of evaluating the responses and probably in the physiological properties of the ORNs. In the experiments on *Drosophila*, the change in the air velocity was provided by varying the air volume delivered during the 5-s pulse. To compensate for the changing molecule arrival rate, the odorant concentration was adjusted to the flow rate. Keeping pulse concentration but varying the volume flow rate led to constant ORN responses. This makes the ORNs “concentration detectors.” The authors did not evaluate the possible effect of the rate of concentration change at the pulse onset slope. Visually, the miniPID signal indicates that a concentration pulse of 50% attained within the first 100 ms of the transient concentration increase a rate of 300%/s (Figure 4 in Zhou and Wilson, 2012). Potentially, such high rates are at the upper limit of sensitivity and therefore variations in the air flow rate ranging between 1.4 and 6.6 m/s did not influence the ORNs responses.

In attempting to assign the ORNs to flux detectors or concentration detectors, the latter is preferable due to their insensitivity to air velocity (Kaissling, 1998). This mechanism was initially applied to CO_2_ receptor neurons, whereas the typical odorant receptors were regarded as flux detectors. Interestingly, the two mechanisms may not be exclusive but instead complement each other in transduction (Rospars et al., 2000; Baker et al., 2012). Note that the odorant detection models are based on rapid odorant uptake, rapid ORN activation and rapid odorant deactivation. Further experiments with slow and continuous concentration changes on different insects should reveal whether the increased sensitivity according to gain control is a widespread coding strategy of encountering fluctuating concentration changes in the olfactory environment. Much work lies still ahead to refine our understanding of how ORNs satisfy the ability to discriminate differences in the rate of concentration changes despite flow velocity-dependent variations in the number of molecules.

## Data Availability

The raw data supporting the conclusions of this manuscript will be made available by the authors, without undue reservation, to any qualified researcher.

## Ethics Statement

All the experiments described in the manuscript were performed with laboratory-reared insects. No special permit was required. After experiments, cockroaches were quickly killed by freezing. All institutional and national guidelines for the care and use of laboratory animals were followed.

## Author Contributions

MH and HT conceived and designed the research, interpreted the results of experiments, and wrote and edited the manuscript. MH and AM performed the experiments and analyzed the data. MH prepared the figures. HT approved the final version of the manuscript.

## Conflict of Interest Statement

The authors declare that the research was conducted in the absence of any commercial or financial relationships that could be construed as a potential conflict of interest.

## References

[B1] Altner H. Loftus R. Schaller-Selzer L. Tichy H. (1983). Modality-specificity in insect sensilla and multimodal input from body appendages. *Fort. Zool.* 28 17–31.

[B2] Atema J. (1996). Eddy chemotaxis and odor landscapes: exploration of nature with animal sensors. *Biol. Bull.* 191 129–138. 10.2307/1543074 29220222

[B3] Baker T. C. Domingue M. J. Myrick A. J. (2012). Working range of stimulus flux transduction determines dendrite size and relative number of pheromone component receptor neurons in moths. *Chem. Senses* 37 299–313. 10.1093/chemse/bjr122 22230170

[B4] Boeckh J. Distler P. Ernst K. D. Hösel M. Malun D. (1990). “Olfactory bulb and antennal lobe,” in *Proceedings of the Chemosensory Information Processing. NATO ASI Series*, (Heidelberg: Springer).

[B5] Burgstaller M. Tichy H. (2011). Functional asymmetries in cockroach ON and OFF olfactory receptor neurons. *J. Neurophysiol.* 105 834–845. 10.1152/jn.00785.2010 21160009 PMC3059178

[B6] Burgstaller M. Tichy H. (2012). Adaptation as a mechanism for gain control in cockroach ON and OFF olfactory receptor neurons. *Eur. J. Neurosci.* 35 519–525. 10.1111/j.1460-9568.2012.07989.x 22304687

[B7] Günther H. (1968). Untersuchungen an Citronenölen mit Hilfe der Gaschromatographie und der Infrarotsprektroskopie. *Dtsch. Lebensm. Rdsch.* 64 104–111.

[B8] Hinterwirth A. Zeiner R. Tichy H. (2004). Olfactory receptor cells on the cockroach antennae: responses to the directions and rate of change in food odour concentration. *Eur. J. Neurosci.* 19 3389–3392. 10.1111/j.0953-816x.2004.03386.x 15217396

[B9] Kaissling K. E. (1998). Flux detectors versus concentration detectors: two types of chemoreceptors. *Chem. Senses* 23 99–111. 10.1093/chemse/23.1.99 9530975

[B10] Keller T. A. Weissburg M. J. (2004). Effects of odor flux and pulse rate on chemosensory tracking in turbulent odor plumes by the blue crab. Callinectes sapidus. *Biol. Bull.* 207 44–55. 10.2307/1543627 15315942

[B11] Moore P. A. Atema J. (1991). Spatial information in the three-dimensional fine structure of an aquatic odor plume. *Biol. Bull.* 181 404–418. 10.2307/1542361 29304679

[B12] Page J. L. Dickman B. D. Webster D. R. Weissburg M. J. (2011). Getting ahead: context-dependent responses to odorant filaments drive along-stream progress during odor tracking in blue crabs. *J. Exp. Biol.* 214 1498–1512. 10.1242/jeb.049312 21490258

[B13] Prah J. D. Sears S. B. Walker J. C. (1995). “Modern approaches to air dilution olfactometry,” in *Handbook of Olfaction and Gustation*, ed. Doty R.-L. (New York, NY: Dekker), 227–255.

[B14] Reidenbach M. A. Koehl M. A. R. (2011). The spatial and temporal patterns of odors sampled by lobsters and crabs in a turbulent plume. *J. Exp. Biology.* 214 3138–3153. 10.1242/jeb.057547 21865526

[B15] Rospars J.-P. Kr̆ivan V. Lánský P. (2000). Perireceptor and receptor events in olfaction. Comparison of concentration and flux detectors: a modeling study. *Chem. Senses* 25 293–311.. 10.1093/chemse/25.3.293 10866988

[B16] Sass H. (1978). Olfactory receptors on the antenna of *Periplaneta*. Response constellations that encode food odours. *J. Comp. Physiol.* 128 227–233. 10.1038/srep27495 27279336 PMC4899716

[B17] Schaller D. (1978). Antennal sensory system of *Periplaneta americana* L. *Cell. Tissue Res.* 191 121–139. 688350 10.1007/BF00223221

[B18] Selzer R. (1981). The processing of a complex food odour by antennal olfactory receptors of *Periplaneta americana*. *J. Comp. Physiol.* 144 509–519. 10.1007/bf01326836

[B19] Selzer R. (1984). On the specificities of antennal olfactory receptor cells of *Periplaneta americana*. *Chem. Senses* 8 375–395. 10.3389/fncir.2017.00032 28529476 PMC5418552

[B20] Shaw P. E. (1979). Review of quantitative analysis of citrus essential oils. *J. Agric. Food Chem.* 27 246–257. 10.1021/jf60222a032

[B21] Tichy H. Hellwig M. (2018). Independent processing of increments and decrements in odorant concentration by ON and OFF olfactory receptor neurons. *J. Comp. Physiol.* 204 873–891. 10.1007/s00359-018-1289-6 30251036 PMC6208657

[B22] Tichy H. Hinterwirth A. Gingl E. (2005). Olfactory receptors on the cockroach antenna signal odour ON and odour OFF by excitation. *Eur. J. Neurosci.* 22 3147–3160. 10.1111/j.1460-9568.2005.04501.x 16367781

[B23] Vickers N. J. (2000). Mechanisms of animal navigation in odor plumes. *Biol. Bull.* 2000 203–212. 10.2307/1542524 10786941

[B24] Vickers N. J. (2006). Winging it: moth flight behavior and responses of olfactory neurons are shaped by pheromone plume dynamics. *Chem. Senses* 31 155–166. 10.1093/chemse/bjj011 16339269

[B25] Webster D. R. Weissburg M. J. (2001). Chemosensory guidance cues in a turbulent chemical odor plume. *Limnol. Oceanogr.* 46 1034–1047. 10.4319/lo.2001.46.5.1034

[B26] Weissburg M. J. (2000). The fluid dynamical context of chemosensory behavior. *Biol. Bull.* 198 188–200. 10786940 10.2307/1542523

[B27] Willis M. A. Avondet J. L. (2005). Odor-modulated orientation in walking male cockroaches *Periplaneta americana*, and the effects of odor plumes of different structure. *J. Exp. Biol.* 208 721–735. 10.1242/jeb.01418 15695764

[B28] Willis M. A. Avondet J. L. Finnell A. S. (2008). Effects of altering flow and odor information on plume tracking behavior in walking cockroaches, *Periplaneta americana* (L.). *J. Exp. Biol.* 211 2317–2326. 10.1242/jeb.016006 18587126

[B29] Zeiner R. Tichy H. (2000). Integration of temperature and olfactory information in cockroach antennal lobe glomeruli. *J. Comp. Physiol.* 186 717–727. 10.1007/s003590000125 11016787

[B30] Zettler E. Atema J. (1999). Chemoreceptor cells as concentration slope detectors: preliminary evidence from the lobster nose. *Biol. Bull.* 198 252–253. 10.2307/1542633 28281804

[B31] Zhou Y. Wilson R. I. (2012). Transduction in drosophila olfactory receptor neurons is invariant to air speed. *J. Neurophysiol.* 108 2051–2059. 10.1152/jn.01146.2011 22815404 PMC3544999

